# The gland localized *CGP1* controls gland pigmentation and gossypol accumulation in cotton

**DOI:** 10.1111/pbi.13323

**Published:** 2020-01-21

**Authors:** Wei Gao, Fu‐Chun Xu, Lu Long, Yang Li, Jun‐Li Zhang, Leelyn Chong, Jose Ramon Botella, Chun‐Peng Song

**Affiliations:** ^1^ State Key Laboratory of Cotton Biology School of Life Science Henan University Kaifeng China; ^2^ State Key Laboratory of Crop Stress Adaptation and Improvement Henan University Kaifeng China; ^3^ School of Agriculture and Food Sciences University of Queensland Brisbane Qld Australia

**Keywords:** MYB transcription factor, GoPGF, CRISPR, secondary metabolites, terpenoids

## Abstract

Pigment glands, also known as black glands or gossypol glands, are specific for *Gossypium* spp. These glands strictly confine large amounts of secondary metabolites to the lysigenous cavity, leading to the glands’ intense colour and providing defence against pests and pathogens. This study performed a comparative transcriptome analysis of glanded versus glandless cotton cultivars. Twenty‐two transcription factors showed expression patterns associated with pigment glands and were characterized. Phenotypic screening of the genes, via virus‐induced gene silencing, showed an apparent disappearance of pigmented glands after the silencing of a pair of homologous MYB‐encoding genes in the A and D genomes (designated as *CGP1*). Further study showed that *CGP1a* encodes an active transcription factor, which is specifically expressed in the gland structure, while *CGP1d* encodes a non‐functional protein due to a fragment deletion, which causes premature termination. RNAi‐mediated silencing and CRISPR knockout of *CGP1* in glanded cotton cultivars generated a glandless‐like phenotype, similar to the dominant glandless mutant *Gl_2_^e^*. Microscopic analysis showed that *CGP1* knockout did not affect gland structure or density, but affected gland pigmentation. The levels of gossypol and related terpenoids were significantly decreased in *cgp1* mutants, and a number of gossypol biosynthetic genes were strongly down‐regulated. CGP1 is located in the nucleus where it interacts with GoPGF, a critical transcription factor for gland development and gossypol synthesis. Our data suggest that CGP1 and GoPGF form heterodimers to control the synthesis of gossypol and other secondary metabolites in cotton.

## Introduction

Pigment glands are specialized cavity structures of *Gossypium* spp. These cavity structures store high concentrations of a wide variety of secondary metabolites, which appear as small dark dots that have also been referred to as black glands (Bell and Stipanovic, 1977). The development of pigment glands involves a cell‐lysigenous process coupled with programmed cell death (Liu *et al.*, 2010). Pigment glands originate from a cluster of gland primordium cells beneath the epidermis, which are characterized by high‐density cytoplasm and large nucleolus (Yatsu *et al.*, 1974). Mature glands consist of a lysigenous cavity, formed via degradation of the central primordium cells, surrounded by thick‐walled cells (Liu *et al.*, 2010; Yatsu *et al.*, 1974). The large amount of secondary metabolites stored in the lysigenous cavities of pigment glands protect plants against pathogens, insects and herbivores, and often have high medicinal value (Kong *et al.*, 2010; Shailendra, 2013). The terpenoid gossypol, produced by members of *Gossypium* spp., is the most studied secondary metabolite stored in pigment glands (Tian *et al.*, 2018). Several studies have suggested that gossypol is mainly synthesized in cotton roots before its transport and storage in mature pigment glands. The pigment glands are considered the main storage structures but not the major synthesis site of secondary metabolites (Smith, 1961, 1962). Most commercial cotton species have typical gland structures, while several wild species such as *G. australe*, *G. stockii* and *G. bickii* have special types (Brubaker, 1996; Kulkarni *et al.*, 2002).

Cotton is the leading commercial crop for the production of natural fibres for the textile industry worldwide. In addition, cotton seeds are an excellent source of edible protein (23%) and oil (21%), are rich in unsaturated fatty acids and have the potential to feed half a billion people globally (Lusas and Jividen, 1987; Sunilkumar *et al.*, 2006). However, the potential of cotton as a food source is limited due to the toxicity of gossypol for humans and other monogastric animals (Zhang *et al.*, 2007). For a long time, breeders have tried to introduce the natural glandless trait to glanded cotton to generate varieties that contain glands in the plant body but that produce glandless seeds to improve the commercial value of cotton seeds while maintaining cotton’s natural resistance to insects and pathogens (Rathore *et al.*, 2012; Sunilkumar *et al.*, 2006). Understanding the key factors that control gland biogenesis and gossypol synthesis is pivotal towards achieving this goal. Sunilkumar *et al. *(2006) used a seed‐specific promoter to silence the limiting enzyme in the synthesis of gossypol, cadinene synthase, using RNAi. This resulted in plants with low gossypol levels in seeds but normal levels in other tissues. Although these genetically modified plants achieved the desired goal, their adoption has been limited due to the current regulations that limit the use of transgenic organisms in some countries (Rathore *et al.*, 2012; Sunilkumar *et al.*, 2006).

Despite a century of extensive studies, little is known about the genetics underlying the biogenesis of cotton glands. Breeding and genetic research have identified six independent loci that control cotton gland formation: *gl_1_*, *gl_2_*, *gl_3_*, *gl_4_*, *gl_5_* and *gl_6_* (Lusas and Jividen, 1987; McMichael, 1960). The six loci regulate gland formation in different cotton tissues, and each locus contains multiple alleles. Loci *Gl_2_* and *Gl_3_* play a major role in gland biogenesis, and the g*l_2_gl_2_*/*gl_3_gl_3_* combination produces a completely glandless phenotype in the tetraploid *G. hirsutum*. However, the presence of the dominant alleles (*Gl_2_* or *Gl_3_*) in any combination results in the appearance of glands with variable distribution in different organs (McMichael, 1960). Alleles *gl_4_* and *gl_5_* reduce gland density, while *gl_1_* and *gl_6_* have similar but weaker effects on gland formation compared with *gl_2_* and *gl_3_* (Lusas and Jividen, 1987). The whole‐plant glandless mutant ‘Bahtim110’ was obtained in 1966 by radiation mutagenesis of the sea‐island cotton ‘Giza45’. Genetic analysis identified the mutant as *Gl_2_^e^*, which is a dominant allele of *Gl_2_* that shows epistatic effect on *Gl_3_* (Afifi *et al.*, 1966). Since then, dozens of glandless varieties have been bred by hybridization and by selecting introgression lines that contain *Gl_2_^e^*; however, the understanding of the molecular basis of gland formation achieved limited progress over the following 50 years. In 2016, Ma et al. used map‐based cloning and identified a basic helix‐loop‐helix (bHLH) transcription factor that controls gland development and was named *Gossypium Pigment Gland Formation* (*GoPGF*). The authors designated the *GoPGF* gene on chr. A12 as *Gl_2_* and the *GoPGF* gene on chr. D12 as *Gl_3_*. Studies on glandless mutants showed that a Val for Ala amino acid substitution at residue 43 of *Gl_2_* results in the dominant *Gl_2_^e^* allele, while single nucleotide insertions into *Gl_2_* and *Gl_3_* introduce premature stop codons and generate the recessive *gl_2_* and *gl_3_* alleles (Ma *et al.*, 2016). Later, Cheng *et al.* (2016) confirmed the identity of *GoPGF* using near‐isogenic lines (NILs) at the *Gl_2_^e^* locus. Comparative transcriptome analysis of glanded and glandless cotton embryos identified three *Cotton Gland Formation* (*CGF*) genes that participate in gland formation (Janga *et al.*, 2019). *CGF2* has a mild effect on gland density, while silencing *CGF1* and *CGF3* resulted in a dramatic reduction in gland numbers. *CGF3* is identical to the previously identified *GoPGF* gene, and extensive mutations in the promoter of A/D subgenomes contributed to the variation of gland phenotypes (Janga *et al.*, 2019).

In this work, we performed comparative transcriptome analysis of several glanded and glandless cultivars, and identified a transcription factor named *Cotton Gland Pigmentation 1* (*CGP1*) involved in the regulation of gland pigmentation but not morphogenesis. Silencing and CRISPR knockout of *CGP1* decrease the accumulation of gossypol and of related terpenoids, as well as colour intensity in glands. Our results advance our understanding of the molecular basis of cotton secondary metabolite synthesis and could have biotechnological applications in the production of cotton seeds without gossypol.

## Results

### Identification of differentially expressed genes in glanded vs glandless cotton cultivars

**Figure 1:**
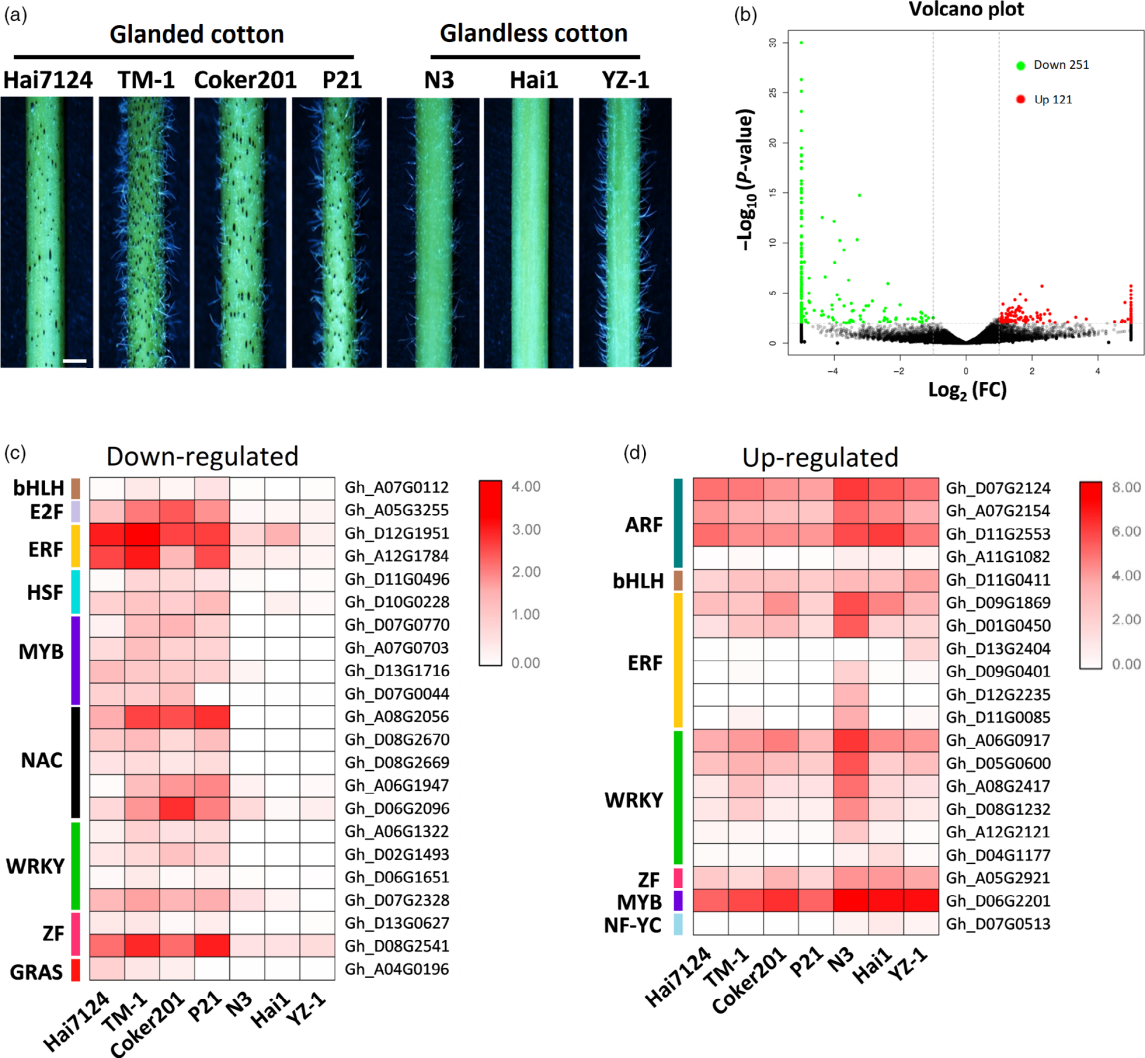
Differentially expressed genes (DEGs) in glanded and glandless cotton. (a) Stems of glanded (‘Hai7124’, ‘TM‐1’, ‘Coker201’ and ‘P21’) and glandless (‘N3’, ‘Hai1’ and ‘YZ‐1’) cotton cultivars, bar = 2 mm. (b) Volcano plot of DEGs in glandless cotton compared with glanded cotton. The green and red dots indicate down‐regulated and up‐regulated genes, respectively. Heat‐map of down‐regulated (c) and up‐regulated (d) transcription factors in glandless cotton compared with glanded cotton. The RPKM values of each gene are shown by a colour gradient from low (white) to high (red).

Young stems of four glanded cultivars (*G. barbadense* L. ‘Hai7124’, *G. hirsutum* L. ‘Coker201’, *G. hirsutum* L. ‘P21’ and *G. hirsutum* L. ‘TM‐1’) and three glandless cultivars (*G. barbadense* L. ‘Hai1’, *G. hirsutum* L. ‘YZ‐1’ and *G. hirsutum* L. ‘N3’) were used for comparative transcriptome analysis (Figure 1a and Figure S1). Among these cultivars, ‘YZ‐1’ is a *gl_2_gl_2_/gl_3_gl_3_* mutant with low *Gl_2_* and *Gl_3_* expression, ‘Hai1’ was produced by introducing *Gl_2_^e^* in sea‐island cotton (Tang *et al.*, 1996), and ‘P21’ and ‘N3’ are NILs at the *Gl_2_^e^* locus (Cheng *et al.*, 2016). A total of 372 deferentially expressed genes (DEGs) were identified via a fold change threshold of ≥2 (*P* value ≤0.01), where 251 genes (67.5%) were down‐regulated and 121 genes (32.5%) were up‐regulated in glandless cotton compared with glanded cotton (Figure 1b and Table S1). The DEGs encompassed most major biological processes with the metabolic and oxidation‐reduction processes being the most enriched groups. Several genes involved in terpene biosynthesis were present among the DEGs (Table S2 and Figure S2).

All three proteins currently known to be involved in cotton pigment gland biology are transcription factors (TFs) (Janga *et al.*, 2019; Ma *et al.*, 2016); therefore, this study focused on the differentially expressed TFs. Within the set of 372 DEGs, 22 TFs were down‐regulated and 20 TFs were up‐regulated in the stems of glandless cotton compared with glanded cotton cultivars (Figure 1c,d and Table S3). The 22 TFs down‐regulated in glandless cultivars are good candidates to have a regulatory role in gland development and underwent further functional characterization.

### Silencing *CGP1* reduces the number of pigmented glands

**Figure 2:**
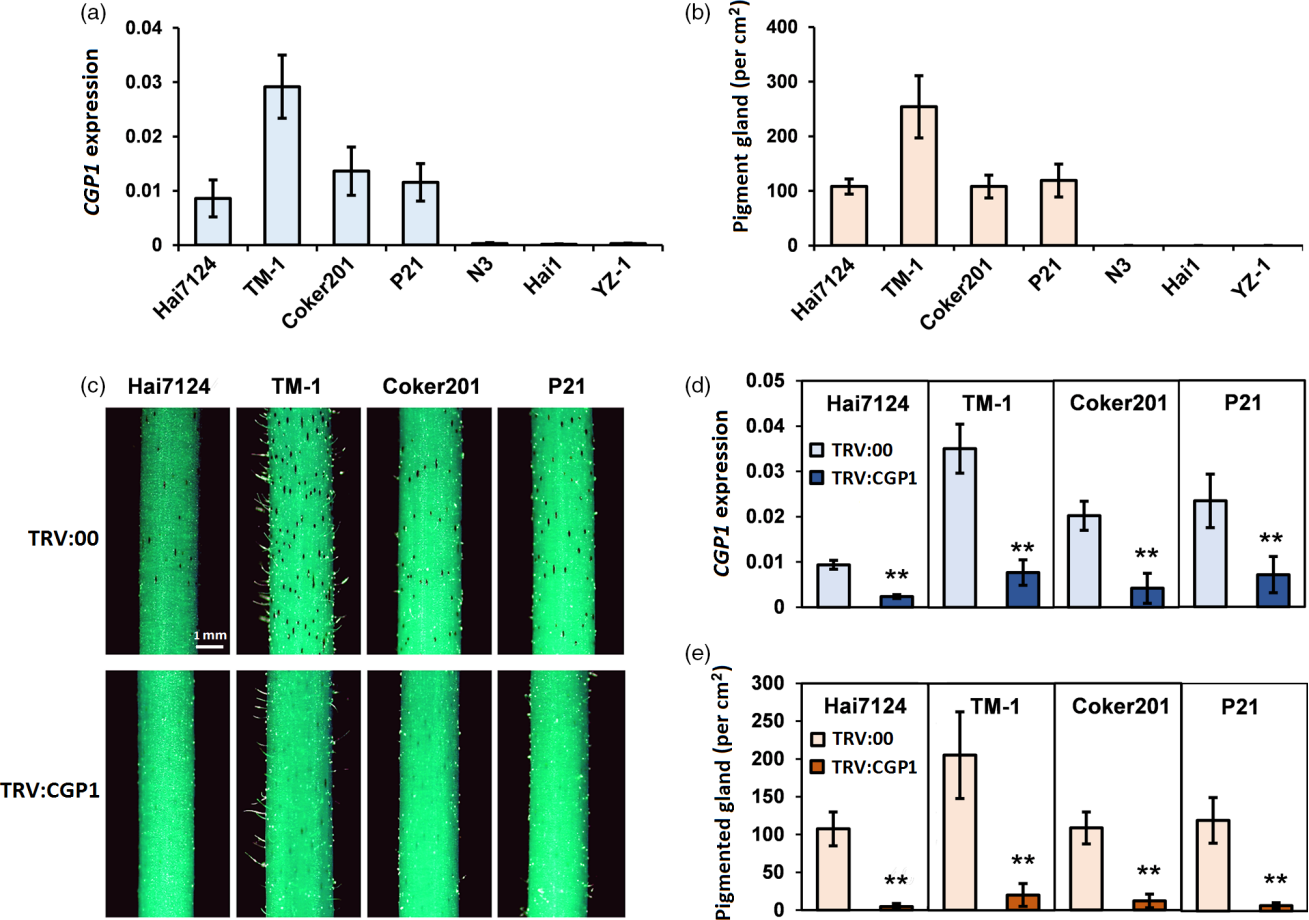
Silencing *CGP1* reduces the number of pigmented glands. (a) *CGP1* expression levels in stems of glanded and glandless cultivars (*n* ≥ 8). (b) Number of pigment glands in stems of glanded and glandless cultivars (*n* ≥ 15). (c) Phenotypes after *CGP1* silencing in stems of glanded cotton cultivars ‘Hai7124’, ‘TM‐1’, ‘Coker201’ and ‘P21’ (TRV:00, empty vector control plants; TRV:CGP1, *CGP1* silencing plants). (d) The expression of *CGP1* in stems of seedlings infiltrated with TRV:00 and TRV:CGP1 (*n* ≥ 8, ** *P* < 0.01, *t*‐test); (e) number of pigmented glands on stems of seedlings infiltrated with TRV:00 and TRV:CGP1 (*n* ≥ 15, ** *P* < 0.01, *t*‐test).

Virus‐induced gene silencing (VIGS) is a rapid and effective method for the silencing of target genes and has been widely used for functional studies in cotton (Gao *et al.*, 2013; Hu *et al.*, 2017). The 22 down‐regulated TFs were individually silenced in glanded cotton seedlings using VIGS, except for several highly homologous genes that were simultaneously silenced using a single VIGS construct. A total of 14 vectors were used to cover the 22 TFs in the cultivar ‘TM‐1’ (Table S3). The silencing efficiency of each VIGS construct was confirmed by quantitative PCR (qPCR) amplification, and the number of pigment glands on the stems of VIGS plants was quantified. Twenty of the silenced TFs did not affect the pigment gland density (Figure S3), while silencing a pair of homologous genes (*Gh_A07G0703*/*Gh_D07G0770*) produced a ‘glandless‐like’ phenotype, which was similar to *Gl_2_^e^* (Figure 2).

Analysis of the *Gh_A07G0703* and *Gh_D07G0770* predicted protein sequences identified them as members of the MYB transcription factor family and was named *CGP1* according to subsequent experimental results*. CGP1* transcript levels were almost undetectable in the three glandless cultivars, while variable expression levels were found in all four glanded cultivars (Figure 2a). Among the glanded cultivars, a very strong correlation was found between *CGP1* expression levels and the density of pigment glands (Figure 2b). To validate the initial results, VIGS‐mediated silencing of *CGP1* was performed in the glanded cultivars ‘Hai7124’, ‘TM‐1’, ‘Coker201’ and ‘P21’ resulting in a strong reduction in visible pigmented glands (Figure 2c–e).

To further validate the function of *CGP1*, transgenic cotton (‘Coker201’) lines containing RNA interference constructs were produced. Fifteen independent transgenic lines with reduced *CGP1* expression were obtained, with five of them containing single T‐DNA insertions were selected for further study according to TaqMan qPCR assays and kanamycin resistance segregation (Tables S4 and S5). qPCR confirmed that *CGP1* transcript levels were significantly lower in the transgenic lines compared with wild‐type (WT) plants (Figure S4a). A decrease in pigmented glands was observed in the transgenic lines, and the intensity of the phenotype was directly linked to the *CGP1* silencing efficiency (Figure S4b,c).

### Upland cotton contains a single functional allele for *CGP1*

**Figure 3:**
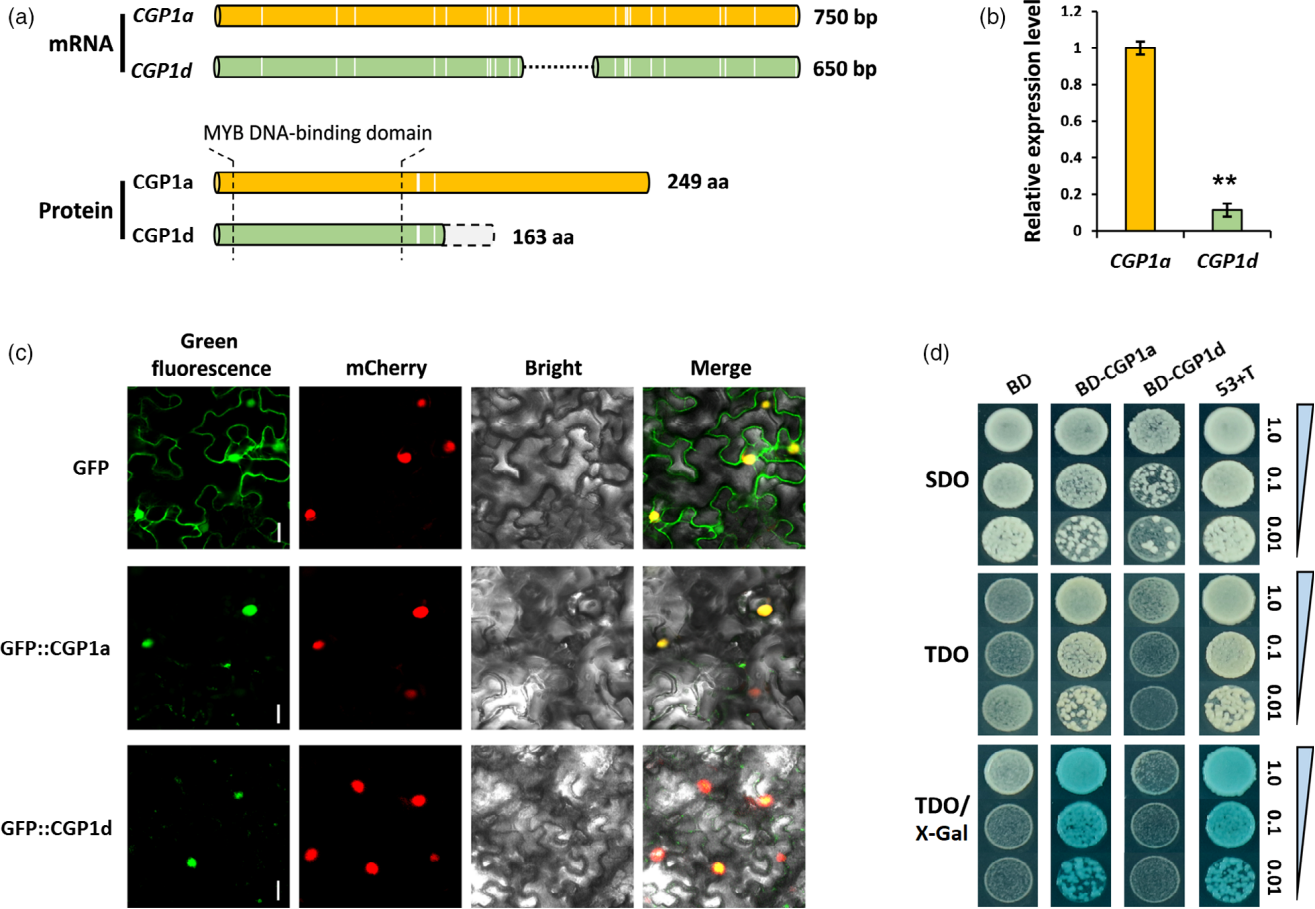
Molecular characterization of *CGP1* in upland cotton. (a) Schematic representation of CGP1 mRNA and protein sequences. The white vertical lines indicate SNPs in *CGP1a* and *CGP1d*; the dotted black line shows the 100‐bp deletion in *CGP1d*; the light grey column boxed with dotted line represents the frameshift of CGP1d protein caused by the 100‐bp deletion; the schematic diagram is shown in scale. (b) Relative expression levels of *CGP1a* and *CGP1d* in cotton stems (n ≥ 5, ** *P* < 0.01, *t*‐test). (c) Subcellular localization of CGP1a and CGP1d in tobacco leaf cells, the red fluorescence of H2B‐mCherry indicates the nucleus, bar = 5 μm. (d) Transcription activity assay of CGP1a and CGP1d using X‐Gal as substrate. SDO (SD/−Trp); TDO (SD/−Ade/−His/−Trp); 53 + T (positive control); BD, empty vector.

The individual *Gh_A07G0703* and *Gh_D07G0770* genes were named *CGP1a* and *CGP1d,* respectively. *CGP1a* and *CGP1d* contain a predicted 750‐bp open reading fragment (ORF) in the reference cotton genome (Zhang *et al.*, 2015), with 97% sequence identity. The 23 single nucleotide polymorphisms between *CGP1a* and *CGP1d* result in 11 amino acid differences (Figure S5). The cDNAs for *CGP1a* and *CGP1d* in ‘TM‐1’ were amplified by RT‐PCR and cloned. Sequencing of the amplification products showed that while the *CGP1a* ORF was identical to the predicted database sequence, *CGP1d* had a 100‐bp deletion resulting in a frameshift at amino acid position 133 (Figure 3a and Figure S6). Consequently, the predicted CGP1d protein contains the MYB DNA‐binding domain but lacks the transcription activation domain. Analysis of the *CGP1a* and *CGP1d* relative expression levels in ‘TM‐1’ stems showed that *CGP1a* transcripts are 11–14 times more abundant than *CGP1d* (Figure 3b).

Transient expression of the CGP1a and CGP1d ORFs fused with the green fluorescent protein (GFP) at the N‐terminus in tobacco epidermal cells produced green fluorescence in the nucleus that co‐localized with the known nuclear protein H2B (fused with the red fluorescent mCherry protein; Martin *et al.*, 2009; Figure 3c). These results suggest that CGP1a and CGP1d are located in the nucleus, and the frameshift did not change the subcellular localization of CGP1d. The transcriptional activation activities of CGP1a and CGP2d were assayed in yeast by fusing the full‐length *CGP1a* and *CGP1d* with the GAL4 DNA‐binding domain (BD). The results showed transcriptional activation of the reporter *GAL4* gene for CGP1a but not for CGP1d (Figure 3d), suggesting that only CGP1a is a functional TF. The 100‐bp fragment absent in *CGP1d* was used in VIGS experiments to specifically silence *CGP1a*. *CGP1a*‐silenced plants showed a similar phenotype to the initial VIGS experiments, which used a sequence common to both genes, that is the absence of pigmented glands (Figure S7). Our results indicate that *CGP1a* is the only functional *CGP1* allele in ‘TM‐1’, controlling the appearance of pigmented glands.

### *CGP1a* is specifically expressed in pigment glands

**Figure 4:**
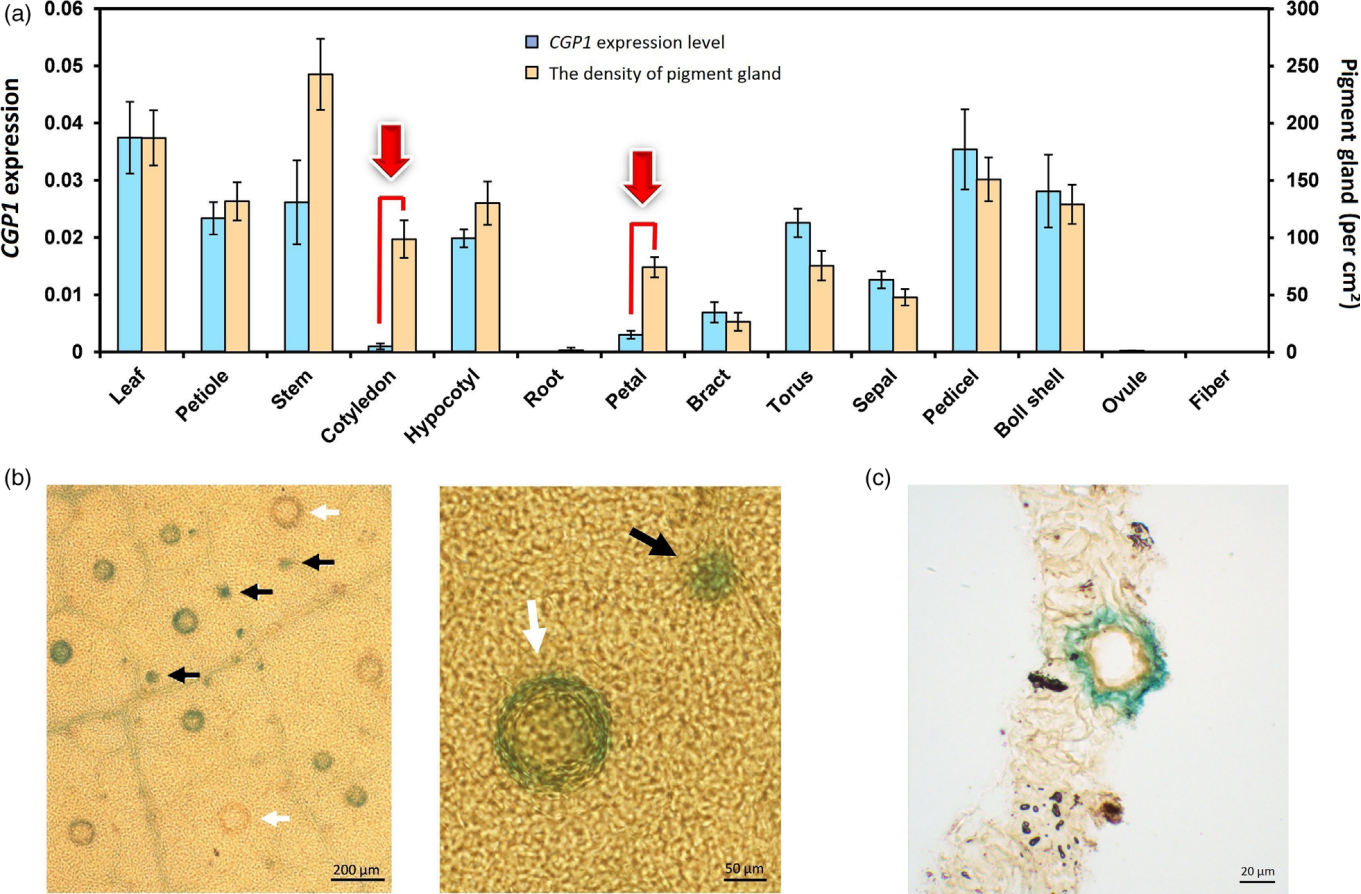
*CGP1* expression analysis. (a) qPCR analysis of *CGP1* expression (blue columns, left ordinate) and number of pigment glands (orange columns, right ordinate) in several vegetative and reproductive organs (*n* ≥ 8); (b) GUS staining of ProCGP1‐GUS transgenic cotton leaves. White arrows indicate mature glands, and black arrows indicate glands at an early developmental stage. (c) Section of ProCGP1‐GUS transgenic cotton leaves. Gland structure in leaves turned blue after GUS staining.

The number of pigment glands varies in different organs of glanded cotton. To determine the relationship between *CGP1* expression levels and pigment glands, the expression levels of *CGP1* were measured in 14 vegetative and reproductive tissues and the density of pigment glands was calculated (Figure 4a). Most vegetative organs, such as young stem, leaf, petiole, hypocotyl and cotyledon, are rich in pigment glands; the main exception is roots. Among reproductive organs, pedicel and boll shell showed high gland densities, while petal, bract, torus and sepal had lower density. Both ovule (5 days postanthesis) and fibre contained no glands. Expression analysis showed a strong correlation between *CGP1* levels and the density of pigment glands with the exception of cotyledons and petals (Figure 4a).

To further study the expression patterns of *CGP1*, a 1.5‐kb promoter fragment upstream of the *CGP1a* initiation codon was cloned upstream of the *β‐glucuronidase* (*GUS*) marker gene in both the glanded cotton variety ‘Coker201’ and the glandless variety ‘YZ‐1’. Several transgenic lines were obtained and used for histochemical staining. GUS staining was not observed in the organs of transgenic glandless ‘YZ‐1’ seedlings (Figure S8), while weak but clear staining was observed in various organs of transgenic ‘Coker201’. Staining in ‘Coker201’ transgenic GUS lines was restricted to pigment glands (Figure 4b,c). Interestingly, not all pigment glands showed GUS staining and most of the stained glands were small and immature.

### Knockout of *CGP1* by CRISPR/Cas9 affects gland pigmentation but not morphogenesis

**Figure 5:**
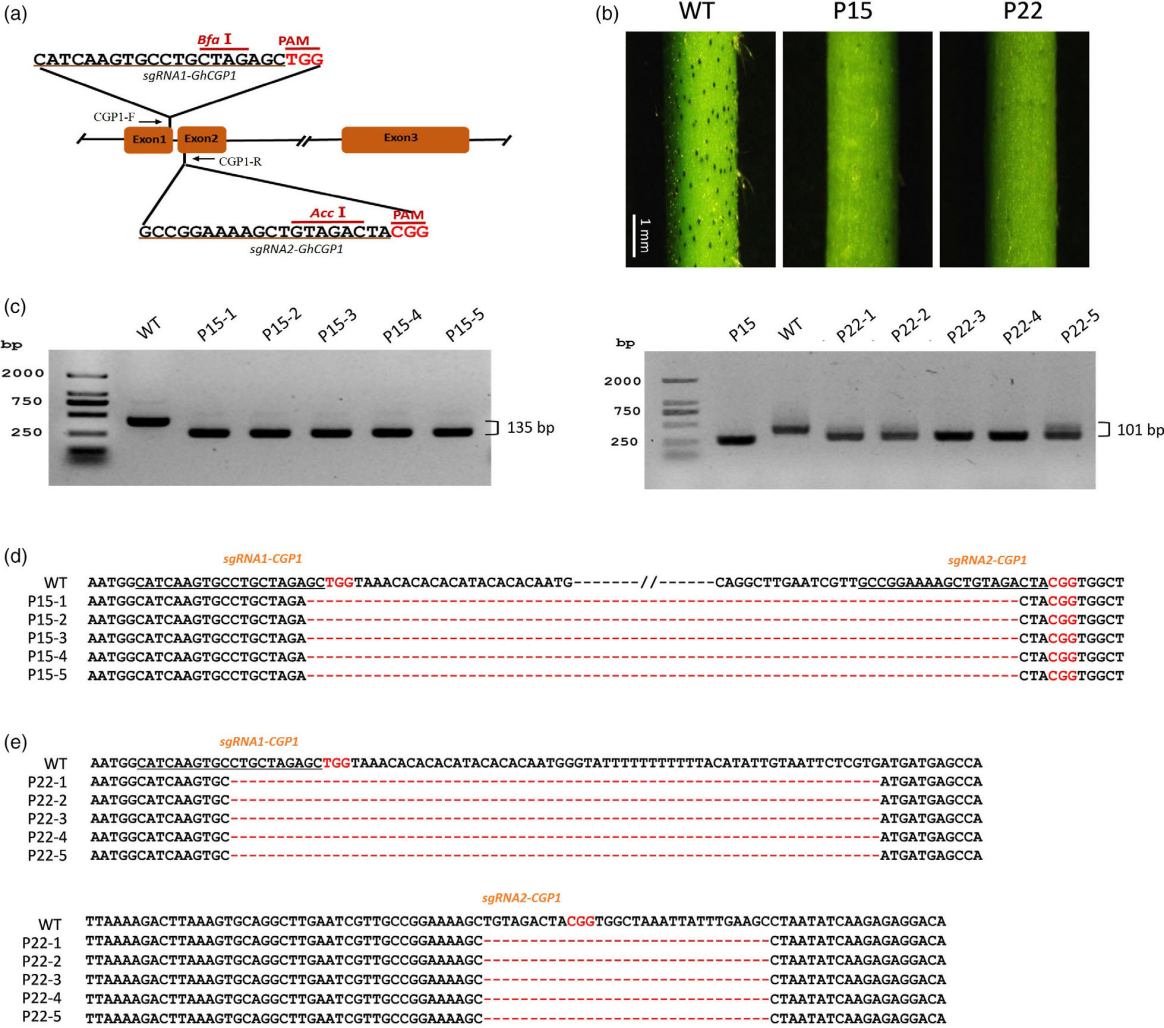
CRISPR/Cas9‐mediated mutation of *CGP1*. (a) Schematic representation of the *CGP1* gene and the two target sites used for CRISPR/Cas9‐mediated mutagenesis. CGP1‐F and CGP1‐R are the forward and reverse primers used for the amplification of the genomic fragment. (b) Phenotypes of *CGP1* knockout lines P15 and P22. (c) PCR amplification of the DNA fragment containing two target sites in five individual P15 and P22 T1 plants. (d, e) Sequencing of the PCR products where deletions are shown as red dashed lines.

CRISPR/Cas9‐mediated knockout of *CGP1* was conducted in the glanded variety ‘Coker201’. To improve the mutagenesis efficiency, two sgRNAs were designed targeting the 1^st^ and 2^nd^ exons, respectively (159 bp away from each other), and were cloned into a single Cas9‐sgRNA cassette (Figure 5a). Twenty‐five independent T_0_ transgenic lines were recovered and two lines (P15 and P22) selected for detailed analysis. After self‐pollination of the T_0_ parentals, five individual T_1_ generation plants for P15 and P22 were used to characterize the genomic locus surrounding the targeted sites. PCR amplification of a genomic fragment containing both targets generated shorter amplicons than those obtained from WT non‐transgenic plants, indicating the presence of deletions (Figure 5c). Sequencing of the amplicons showed that all five P15 seedlings contained a 135‐bp deletion between the two targets (Figure 5d), while P22 seedlings had deletions at both targets (70 and 30 bp) (Figure 5e). As shown in Figure 5b, both P15 and P22 had an almost complete absence of pigmented glands, which is consistent with the VIGS silencing results and confirms the important role of CGP1 in gland biology.

**Figure 6:**
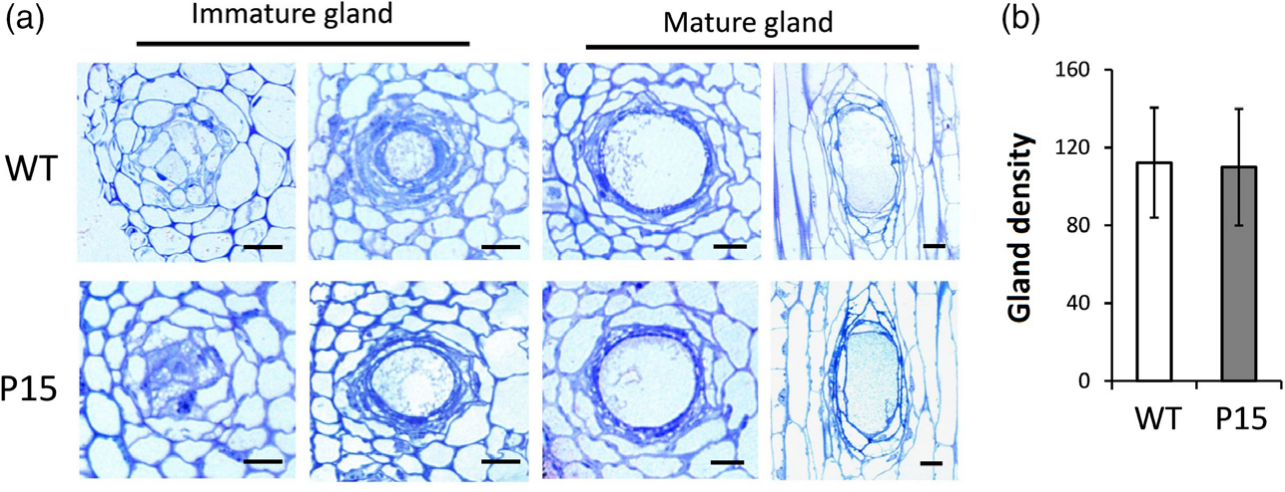
Knockout of *CGP1* does not affect pigment gland structure or density. (a) Pigment glands at different developmental stages in WT and *cgp1* mutant (P15) stems. Left panel shows the cross sections of immature glands; right panel shows the cross and vertical sections of mature glands. bar = 50 μm. (b) Gland density on stems of WT and *cgp1* mutant (P15) plants (*n* ≥ 8).

Detailed structural analyses were conducted by performing 50 serial slices in a 1‐cm stem section of a *cgp1* mutant (P15) and WT. Optical microscopy observations revealed the presence of immature and mature glands in P15, and no obvious differences in gland structure were found between P15 and WT (Figure 6a). Pigment gland numbers were calculated using the slide sets, and the results showed no significant differences between WT and P15 (Figure 6b).

### CGP1 regulates the accumulation of gossypol and related terpenoids

**Figure 7:**
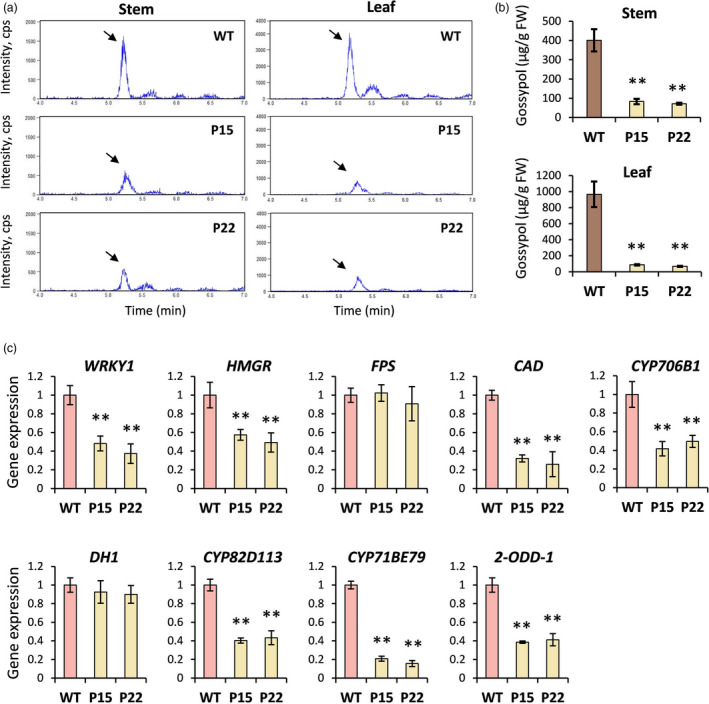
Analysis of gossypol content and synthesis in WT and *cgp1* mutants. (a) LC‐ESI‐MS/MS analysis of gossypol in stems and leaves of WT and *cgp1* mutants (P15 and P22). The gossypol peak is marked with a black arrow. (b) Gossypol content in stems and leaves of WT and *cgp1* mutants (P15 and P22) (*n* ≥ 15, ** *P* < 0.01, *t*‐test). (c) Relative expression levels of gossypol biosynthetic genes in stems of WT and the *cgp1* mutants (P15 and P22) (*n* ≥ 8, ** *P* < 0.01, *t*‐test).

The results of the microscopic studies clarified that the apparent lack of pigmented glands observed in the mutant phenotypes was caused by the absence of pigments. Gossypol has been reported to be one of the major stored metabolites in pigment glands (Tian *et al.*, 2018). The gossypol content was quantified in the stem and leaf tissues of WT and *cgp1* mutants (P15 and P22) using LC‐ESI‐MS/MS (Figure 7a). MS analysis of the peaks (marked with black arrows in Figure 7a) identified the compound as gossypol. The gossypol contents in leaves of P15 and P22 mutants were 10 times lower than in WT, while stems of P15 and P22 mutants contained four times lower levels of gossypol than WT (Figure 7b). Expression analyses showed that most of the gossypol biosynthesis genes were down‐regulated in *cgp1* mutants compared with WT (Figure 7c). Gossypol content was also measured in plants subjected to VIGS‐mediated silencing of *CGP1*. Silencing using the TRV:CGP1 and TRV:CGP1a vectors resulted in a strong reduction in gossypol levels compared with control plants infiltrated with TRV:00 (Figure S9). Furthermore, levels of related terpenoids from the gossypol biosynthetic pathway were measured using HPLC (Janga *et al.*, 2019; Stipanovic *et al.*, 1988) in the CRISPR lines. The levels of hemigossypolon (HGQ) and heliocides (H1, H2 and H3) were strongly reduced in P15 and P22 *cpg1* mutants compared with WT (Figure S10). These results suggest that CGP1 plays an important role in the control of terpenoid accumulation.

### CGP1a interacts with GoPGF in the nucleus

**Figure 8:**
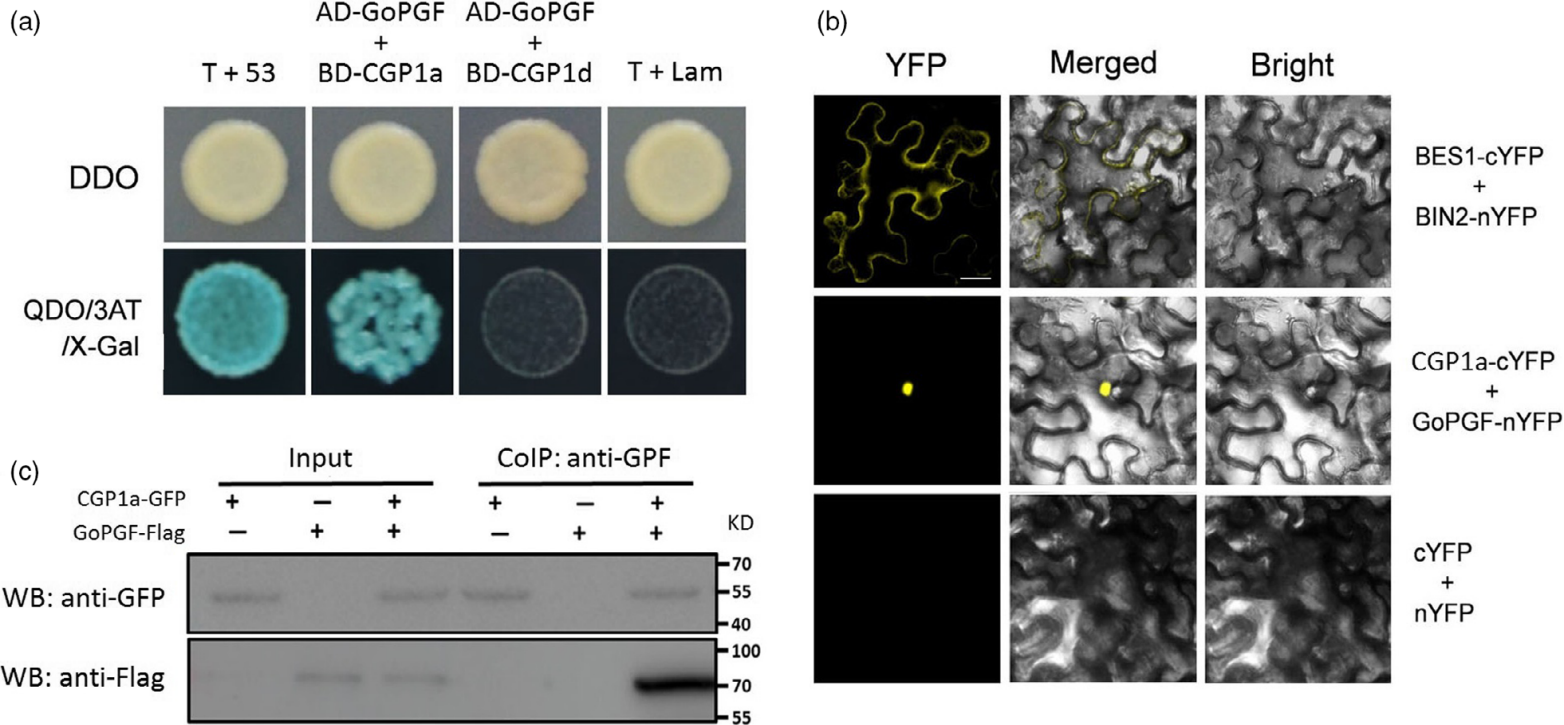
CGP1a interacts with GoPGF in the nucleus. (a) Yeast two‐hybrid assays showing interaction between PGF2a and GoPGF but not between PGF2d and GoPGF. Yeast growth and X‐Gal staining indicate interaction. (b) BiFC assays showing interaction between PGF2a and GoPGF in nuclei of tobacco epidermal cells. PGF2a‐cYFP and GoPGF‐nYFP were co‐expressed in tobacco leaves for the interaction assay. BES1‐cYFP and BIN2‐nYFP were used as positive control; bar = 10 μm. (c) Co‐IP assays of CGP1a and GoPGF. CGP1a‐GFP, GoPGF‐Flag or CGP1a‐GFP/GoPGF‐Flag were expressed in tobacco leaves. The Co‐IP experiment was performed with anti‐GFP affinity gel resin. The isolated proteins were analysed by immunoblotting with anti‐GFP antibody for the detection of CGP1a and with anti‐Flag antibody for the detection of GoPGF.

To identify potential CGP1 interactions in cotton, CGP1a was used as bait to screen a yeast two‐hybrid (Y2H) library. Multiple putative CGP1a interactors were identified in the screening, including a range of TFs, RNA‐binding proteins and protein kinases. GoPGF, a bHLH transcription factor essential in pigment gland biogenesis (Ma *et al.*, 2016), was identified as one of the CGP1a‐interacting proteins. To verify this interaction, GoPGF was cloned in the vector pGADT7 (AD‐GoPGF), while CGP1a/d was cloned in pGBKT7 (BD‐CGP1a/d). Yeast colonies containing AD‐GoPGF plus BD‐CGP1a grew in the presence of 3AT, and X‐Gal activity was observed, supporting the interaction between GoPGF and CGP1a. CGP1d did not interact with GoPGF in yeast cells, likely due to the deletion of the interaction domain caused by a frameshift mutation (Figure 8a). In vivo assays using bimolecular fluorescence complementation (BiFC) and co‐immunoprecipitation (Co‐IP) were conducted to further confirm the interaction between CGP1a and GoPGF. For the BiFC assays, CGP1a was fused to C‐terminal YFP (CGP1a‐cYFP), while GoPGF was fused to N‐terminal YFP (GoPGF‐nYFP). Yellow fluorescence was found in the nucleus when CGP1a‐cYFP and GoPGF‐nYFP were co‐expressed in tobacco epidermal cells, indicating that CGP1a interacts with GoPGF in the cell nucleus in vivo (Figure 8b). For Co‐IP assays, *A. tumefaciens* strains carrying the CGP1a‐GFP and GoPGF‐Flag were co‐expressed in tobacco leaves, and the CGP1a‐GFP or GoPGF‐Flag expressed in leaves alone was used as negative control. The anti‐GFP and anti‐Flag antibodies detected proteins with the expected sizes for CGP1a and GoPGF, respectively. Analysis of immunoprecipitated proteins with Flag antibodies indicated that CGP1a‐GFP was capable of pulling down GoPGF‐Flag (Figure 8c). These results demonstrate that CGP1a and GoPGF can form heterodimers in the nucleus indicating a role for GoPGF, in combination with CGP1, in the control of terpenoid accumulation.

## Discussion

**Figure 9:**
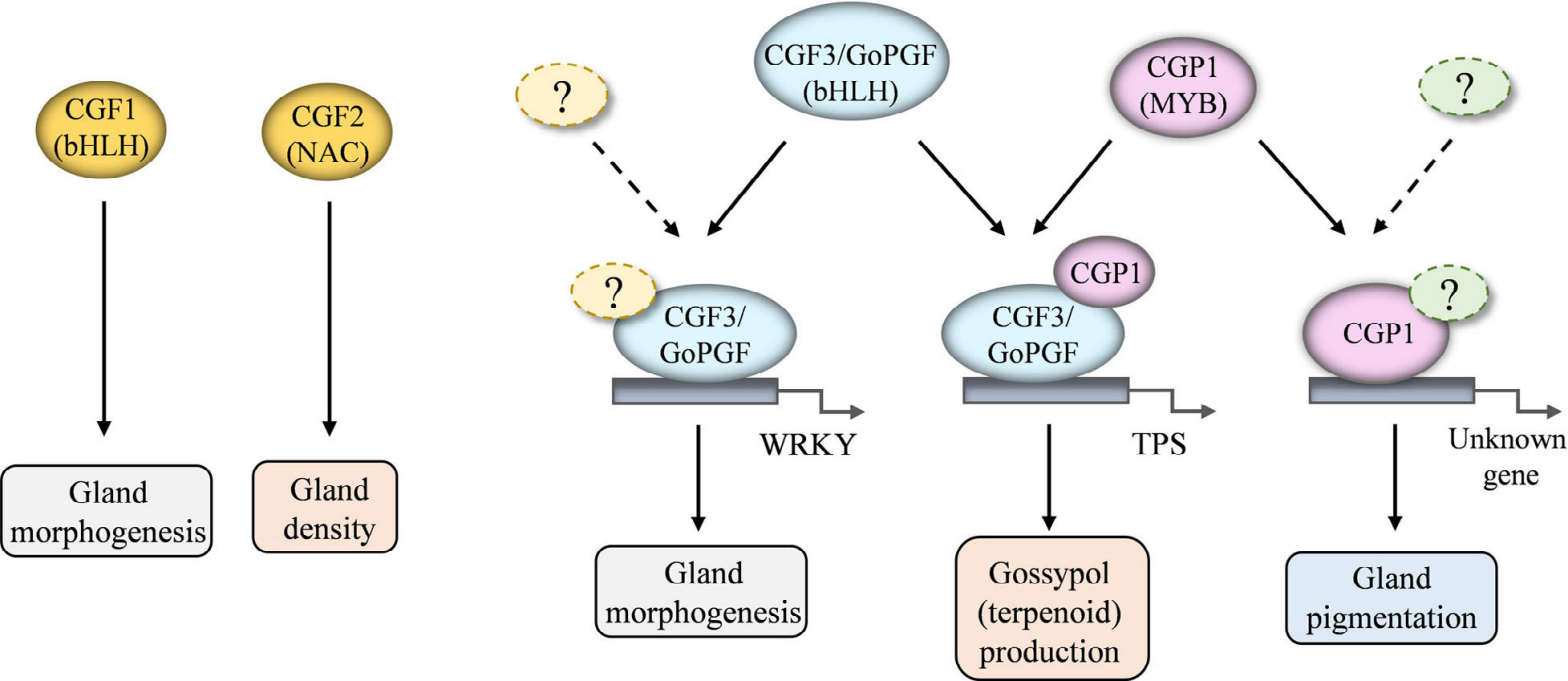
Schematic model illustrating the proposed functions of CGP1, GoPGF and CGFs in cotton gland morphogenesis and pigmentation.

Considering the importance of pigment glands for cotton, knowledge about their biogenesis as well as the secondary metabolites they accumulate is critical for the improvement of this important crop (Janga *et al.*, 2019; Ma *et al.*, 2016; Tian *et al.*, 2018). Nevertheless, the difficulties involved in the production of new glandless mutants and the map‐based cloning of the corresponding loci have so far hindered progress in this field. The recent cloning and characterization of *GoPGF* (Ma *et al.*, 2016) as well as several *CGF* genes (Janga *et al.*, 2019) provided critical insights into the development of pigment glands. GoPGF/CGF3 controls both gland morphogenesis and gossypol synthesis, CGF1 shows similar functions to GoPGF/CGF3, and CGF2 regulates the density of pigment glands (Figure 9). Silencing *GoPGF* abolished pigment gland development in cotton and resulted in almost undetectable gossypol levels (Janga *et al.*, 2019; Ma *et al.*, 2016). Interestingly, although initial observations indicated that silencing and knockout of *CGP1* in glanded cotton produce a similar phenotype to the *gopgf* mutant, detailed analyses showed that *cgp1* mutants had a normal gland structure and WT gland numbers, thus ruling out a role for *CGP1* in gland morphogenesis. The apparent absence of glands in *cgp1* plants was instead due to the lack of coloured pigments. Our results show that *CGP1* knockout leads to down‐regulation of multiple gossypol biosynthetic genes and a dramatic reduction in gossypol levels. Gland formation seems to be independent from gossypol synthesis since transgenic cotton lines with low gossypol levels (due to silencing of the key biosynthetic gene *CYP706B1*) showed normal gland development (Ma *et al.*, 2016).

Ma *et al. *(2016) provided critical evidence suggesting that GoPGF independently regulates gland morphogenesis and gossypol synthesis by binding the promoters of WRKYs and terpene synthases (TPSs), respectively. CGP1 is a MYB TF that regulates gossypol accumulation but not gland morphogenesis. This study proves that CGP1 has transcriptional activity and interacts with GoPGF in the nucleus. MYB proteins tend to form homo‐ and heterodimers to increase affinity and specificity for DNA targets (Dubos *et al.*, 2010; Lu *et al.*, 2009). It is therefore tempting to speculate that CGP1 and GoPGF form heterodimers to regulate the synthesis of gossypol, and perhaps other terpenoids, but not glandular development (Figure 9). Nevertheless, while *GoPGF* is widely expressed throughout cotton plants, the levels of *CGP1* in roots are very low, suggesting that, in roots, GoPGF could either form homodimers or dimerise with other transcription factors. Yeast one‐hybrid assays have shown that GoPGF can bind to the G‐box motif present in the promoters of many WRKYs and TPSs (Ma *et al.*, 2016). It would be interesting to study whether the presence of CGP1 increases the affinity or the in vivo transcription activation of the target genes. It is important to point out that while *GoPGF* knockout leads to an almost complete lack of gossypol, *cgp1* mutants still show residual gossypol levels, which suggests that CGP1 plays an important but not essential role in gossypol regulation.

In addition to gossypol, a number of secondary metabolites are strictly confined to the glands, conferring their characteristic intense colour (Bell and Stipanovic, 1977). Therefore, the lack of pigmentation observed in the *cgp1* mutants strongly suggests that CGP1 controls the synthesis of secondary metabolites other than gossypol. Although the presented expression studies showed that *CGP1* is expressed in most tissues, the GUS staining of immature and mature glands of transgenic promoter lines is intriguing and indicates increased secondary metabolite synthesis activity during this developmental stage.

Gossypol‐free seeds are a highly desirable trait that increases the value of commercial cotton varieties for the utilization of both oil and protein from cottonseeds. Biotechnological manipulation of CGP1 has the potential to increase the defence against pests and pathogens in aerial tissues while either reducing the gossypol content in seeds for food purposes. The cloning and characterization of CGP1 both provide new opportunities to study gland‐contained metabolites and their functions in cotton.

## Experimental procedures

### Cotton materials and growth conditions

The cotton seeds were immersed in water for 2 h and germinated in a high humidity environment at 28 °C for 36 h in the dark. Well‐germinated seeds were planted in the soil for growth at 28 °C (16‐h light and 8‐h dark) in a glasshouse.

### Transcriptome analysis

Total RNA was isolated from the stems of 3‐week‐old seedlings of both glanded and glandless cotton cultivars using the Aidlab RNA extraction kit (Aidlab, Beijing, China). Following quality evaluation, the total RNA was used for cDNA library construction. The resulting cDNA library was then sequenced using the Illumina HiSeqTM 2000 sequencing system. The RNA‐seq raw data were transformed, filtered, and mapped to the *G. hirsutum* genome (http://mascotton.njau.edu.cn/info/1054/1118.htm) following our previously described method (Long *et al.*, 2019). For DEGs analysis, a threshold of fold change ≥2 and a *P* value ≤0.01 were used. Gene annotation was performed using BlastX based on the *G. hirsutum* genome sequence. GO enrichment analysis was performed using Blast2GO (https://www.blast2go.com/).

### PCR amplification

First‐strand cDNA was synthesized using the M‐MLV reverse transcript system (Promega, Beijing, China). PCR amplification of the *CGP1* gene sequence and the promoter sequence were performed with high‐fidelity DNA polymerase Phanta Master Mix (Vazyme, Nanjing, China). qPCR analyses were performed, following a previously described protocol (Gao *et al.*, 2018) using the cotton *Ubiquitin 7* gene (accession: DQ116441) as internal reference.

### Subcellular localization

The coding sequence (CDS) of *CGP1a/d* was inserted into the pK7FWGF2,0 vector to generate the p35S‐GFP::CGP1a/d construct. p35S‐GFP was used as positive control. The construct p35S‐H2B::mCherry was co‐expressed with p35S‐GFP or p35S‐GFP::CGP1a/d to label the nucleus. All constructs were introduced into *A. tumefaciens* for infiltration of tobacco leaves (*Nicotiana benthamiana*). The green and red fluorescence signals were observed using a confocal microscope (Leica, Wetzlar, Germany) 48 h after *A. tumefaciens* infiltration.

### Promoter analysis and GUS staining

The 1.5‐kb fragment upstream of the *CGP1a* transcriptional start site was cloned from ‘TM‐1’ DNA and inserted into the pKGWFS7,0 vector to build the construct ProCGP1‐GUS that expresses GUS derived by the *CGP1a* promoter. This construct was introduced into both glanded cotton *G. hirsutum* L. ‘Coker201’ and glandless cotton *G. hirsutum* L. ‘YZ‐1’ by the *A. tumefaciens*‐mediated stable transformation system (Jin *et al.*, 2006). The GUS staining of tissues was performed following previously published procedures (Deng *et al.*, 2012). Stained samples were cut into sections (Yamaguchi *et al.*, 2010), and images were taken with a light microscope (OLYMPUS IX73, Tokyo, Japan).

### Gene silencing

The vector of the VIGS system was constructed using a previously published method (Gao *et al.*, 2013). Cotyledons of 8‐day‐old cotton seedlings were infiltrated with *A. tumefaciens*, containing VIGS vectors. After 2 weeks, the number of pigmented glands was calculated on the stems of VIGS plants. The stems were harvested for a silencing efficiency analysis. The VIGS experiment was repeated three times. For each repeat, 15 plants were included to silence each target gene.

To generate transgenic cotton silencing *CGP1* with stable transformation, the RNAi vector was introduced into *G. hirsutum* L. ‘Coker201’ (Deng *et al.*, 2012; Jin *et al.*, 2006). Regenerated cotton plants were subjected to antibiotic screening and silencing efficiency analysis by qPCR to select effective transgenic lines with decreased *CGP1* expression. To analyse T‐DNA insertion copies in T_0_ plants, genomic DNA was extracted and TaqMan qPCR was performed. The *UBC1* and *NPTII* were amplified as reference gene and target gene, respectively (Yi *et al.*, 2008). The T_0_ plants with single T‐DNA insertion were self‐pollinated. The seeds of each T_0_ plant were used for the Kanamycin resistance segregation by germinating seeds in medium that contained Kanamycin. The segregation ratios were calculated.

### CRISPR/Cas9‐mediated knockout

The full‐length DNA sequence of *CGP1* was analysed using an online toolkit for CRISPR‐based genome editing (http://skl.scau.edu.cn/). Two putative target sites were selected for sgRNA design, and the designed sgRNAs were assembled into the pYLCRISPR/Cas9 vector (Ma *et al.*, 2015; Wang *et al.*, 2018). Next, constructed methods were performed using a previously reported method (Gao *et al.*, 2017). The stable transformation of *G. hirsutum* L. ‘Coker201’ was further conducted to generate transgenic cotton (Jin *et al.*, 2006). Genomic DNA was extracted from the transgenic line via a DNA extraction kit (Tiangen Biotech, Beijing, China). Furthermore, the primers (CGP1‐F and CGP1‐R) were designed to amplify the fragment across both target sites in DNA to detect the fragment deletion of *CGP1*. The edited gene bands were cloned into a TA‐cloning vector and were subjected to target sequencing (Sangon Biotech, Shanghai, China).

### Light microscopy

Fresh stems of cotton were cut into pieces of 2 mm^2^ and were fixed in a fixative solution (2.5% glutaraldehyde, 0.1 m phosphate buffer, pH 7.0) at 4 °C for 12 h before being embedded in Spurr’s resin. 1‐µm sections from embedded tissues were cut with a microtome (Leica EM UC7) and were stained with toluidine blue for imaging with a light microscope (OLYMPUS IX73).

### Measurement of gossypol and other related terpenoids

Gossypol was extracted from stems or leaves of cotton seedlings as previously described (Tian *et al.*, 2018). For gossypol measurement, supernatants extracted from cotton tissues were filtered using a nylon filter and were then diluted 20 times using methanol for LC‐ESI‐MS/MS (AB Sciex 4000 QTRAP, Boston). Other related terpenoids were isolated and identified as described by Janga *et al. *(2019) and Stipanovic *et al. *(1988). In brief, leaves were freeze‐dried and ground into powder using liquid nitrogen. After extraction with acetonitrile: water: phosphoric acid (80:20:0.1) solution, the obtained extract was analysed using HPLC (Waters e2695, Milford).

### Transcriptional activation assay

The CDS of *CGP1a/d* was fused with the GAL4 DNA BD in the pGBKT7 vector to generate BD‐CGP1a/d. The plasmid pGBKT7‐53 was used as positive control. Each vector was transformed into the Y2H gold yeast strain and plated on SD/‐Trp (SDO) medium for positive selection. Dilutions of yeast clones were then plated onto SD/‐Trp‐His‐Ade (TDO) and SD/‐Trp‐His‐Ade/X‐Gal media for subsequent transcriptional activation assays. Images were taken after incubation on the medium for 3 days.

### Y2H assays

The Y2H assays were conducted as described in the manufacturer’s instructions of Match‐maker Gold Yeast Two‐Hybrid System (Clontech, Mountain View). The BD‐CGP1a construct was produced as bait and was transformed into the yeast strain Y2H. A cotton cDNA library, prepared from different tissues, was used for interaction protein screening. To confirm the observed interactions, AD‐GoPGF and BD‐CGP1d were constructed. Mating between yeast strains that contained AD or BD constructs was performed, and the resultant strains were plated onto amino acid‐deficient medium for screening. After SD‐Trp‐Leu (DDO) screening, positive colonies were plated onto SD‐Trp‐Leu‐His‐Ade (QDO) supplemented with X‐Gal for further validation. Media supplemented with 10 mm of 3‐aminotriazole (3‐AT) were used to remove auto‐activation. Images were taken 3 days after inoculation on the media.

### BiFC and Co‐IP assays in tobacco leaves

For BiFC assays, the CDS of both *CGP1a* and *GoPGF* was individually cloned into pXY104 (cYFP) and pXY106 (nYFP), respectively. The generated constructs of CGP1a‐cYFP and GoPGF‐nYFP were transformed into the *A. tumefaciens* strain GV3101 and were then co‐expressed in the *N. benthamiana* leaves via *Agrobacterium* infiltration. BES1‐cYFP and BIN2‐nYFP were co‐expressed as positive control (Hao *et al.*, 2016). After infiltration for 3 days, yellow fluorescence signals were detected with a confocal microscope (Leica).

For Co‐IP assays, the CDS of *CGP1a* and *GoPGF* was constructed into the pGWB451 and pHB‐Flag, respectively. *A. tumefaciens* carrying CGP1a‐GFP, GoPGF‐Flag or CGP1a‐GFP/GoPGF‐Flag were infiltrated into *N. benthamiana* leaves. After 48 h, the infiltrated leaves were collected for protein extraction. Total proteins were extracted using extraction buffer (50 mm Tris‐HCl, pH 7.5, 150 mm NaCl, 0.5% Triton X‐100, 5% glycerol, 1 mm DTT, 1% protease inhibitor cocktail). Anti‐GFP and anti‐Flag antibodies were used to immunoprecipitate CGP1a‐GFP and GoPGF‐Flag proteins, respectively, and then, the co‐immunoprecipitated proteins were detected. Further procedures were conducted according to the instructions for the Dynabeads Co‐Immunoprecipitation kit (Invitrogen, Carlsbad).

## Author contributions

WG and JRB analysed and interpreted data and wrote the manuscript. FCX, YL and JLZ performed the experiments. LL performed the cotton stable transformation. LC reversed the manuscript. CPS designed the study and supervised all of work. All authors read and approved the final manuscript.

## Competing interests

The authors declare that they have no competing interests.

## Supporting information


**Figure S1** Phenotypes of glanded and glandless cotton cultivars.
**Figure S2** GO analysis of DEGs in glandless cotton compared with glanded cotton.
**Figure S3** Functional characterization of candidate TFs by VIGS in ‘TM‐1’.
**Figure S4** RNAi‐mediated silencing of *CGP1* in stably transformed cotton.
**Figure S5** Genome database sequences of CGP1a and CGP1d.
**Figure S6** Cloned CGP1a and CGP1d sequences.
**Figure S7** Specific silencing of *CGP1a* in cotton.
**Figure S8** GUS staining of the glandless cotton cultivar ‘YZ‐1’ transformed with the ProCGP1‐GUS construct (a, fruit‐bearing branch; b, bud; c, stem; d, pedicel; e, bract; f, leaf), bar = 5 mm.
**Figure S9** Gossypol content in VIGS‐silenced plants.
**Figure S10** Levels of gossypol‐related terpenoids in WT and *cgp1* mutants.

**Table S1** DEGs of glandless cotton compared with glanded cotton.
**Table S2** GO classification of the identified DEGs.
**Table S3** Differentially expressed TFs in glandless cotton compared with glanded cotton.
**Table S4** T‐DNA insertion copies in T_0_ transgenic plants deduced by qPCR.
**Table S5** Separation ratio of T_1_ seeds of RNAi lines selected by kanamycin.
**Table S6** Primers used in this study.
