# Supplementary material for: The gland localized CGP1 controls gland pigmentation and gossypol accumulation in cotton
Source: Plant Biotechnol J. 2020 Jan 21;18(7):1573–84. doi: 10.1111/pbi.13323 (PMC7292540; doi:10.1111/pbi.13323)
Supplement: Supplementary file 1 — Figure S1 Phenotypes of glanded and glandless cotton cultivars. Figure S2 GO analysis of DEGs in glandless cotton compared with glanded cotton. Figure S3 Functional characterization of candidate TFs by VIGS in ‘TM‐1’. Figure S4 RNAi‐mediated silencing of CGP1 in stably transformed cotton. Figure S5 Genome database sequences of CGP1a and CGP1d. Figure S6 Cloned CGP1a and CGP1d sequences. Figure S7 Specific silencing of CGP1a in cotton. Figure S8 GUS staining of the glandless cotton cultivar ‘YZ‐1’ transformed with the ProCGP1‐GUS construct (a, fruit‐bearing branch; b, bud; c, stem; d, pedicel; e, bract; f, leaf), bar = 5 mm. Figure S9 Gossypol content in VIGS‐silenced plants. Figure S10 Levels of gossypol‐related terpenoids in WT and cgp1 mutants. [file PBI-18-1573-s001.docx]

**
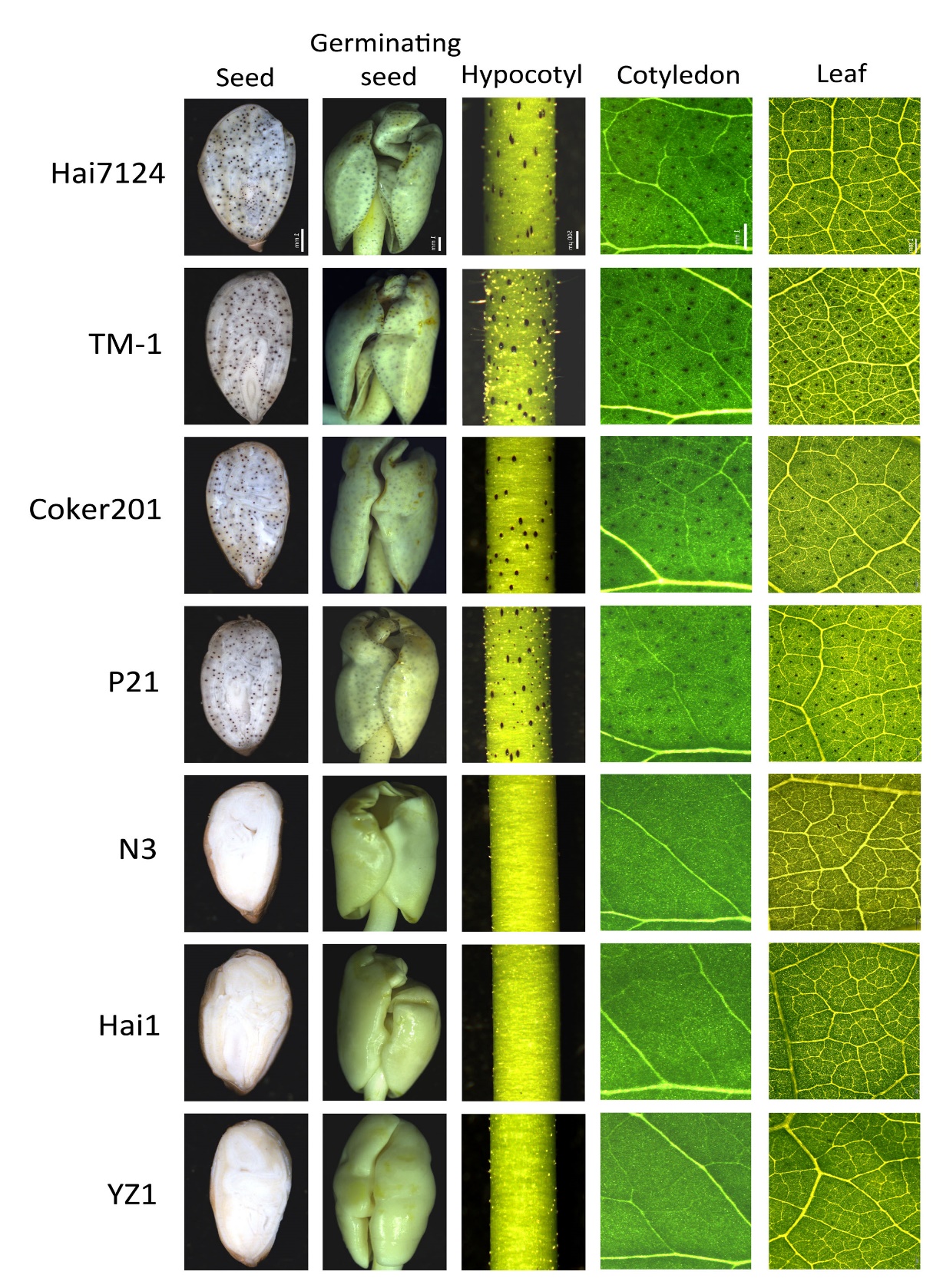
**

**Figure S1** Phenotypes of glanded and glandless cotton cultivars. Microscopic images of pigment gland on seeds, germinating seeds, hypocotyls, cotyledons, and true leaves of four glanded (‘Hai7124’, ‘TM-1’, ‘Coker201’, and ‘P21’) and three glandless (‘N3’, ‘Hai1’, and ‘YZ-1’) cotton cultivars.


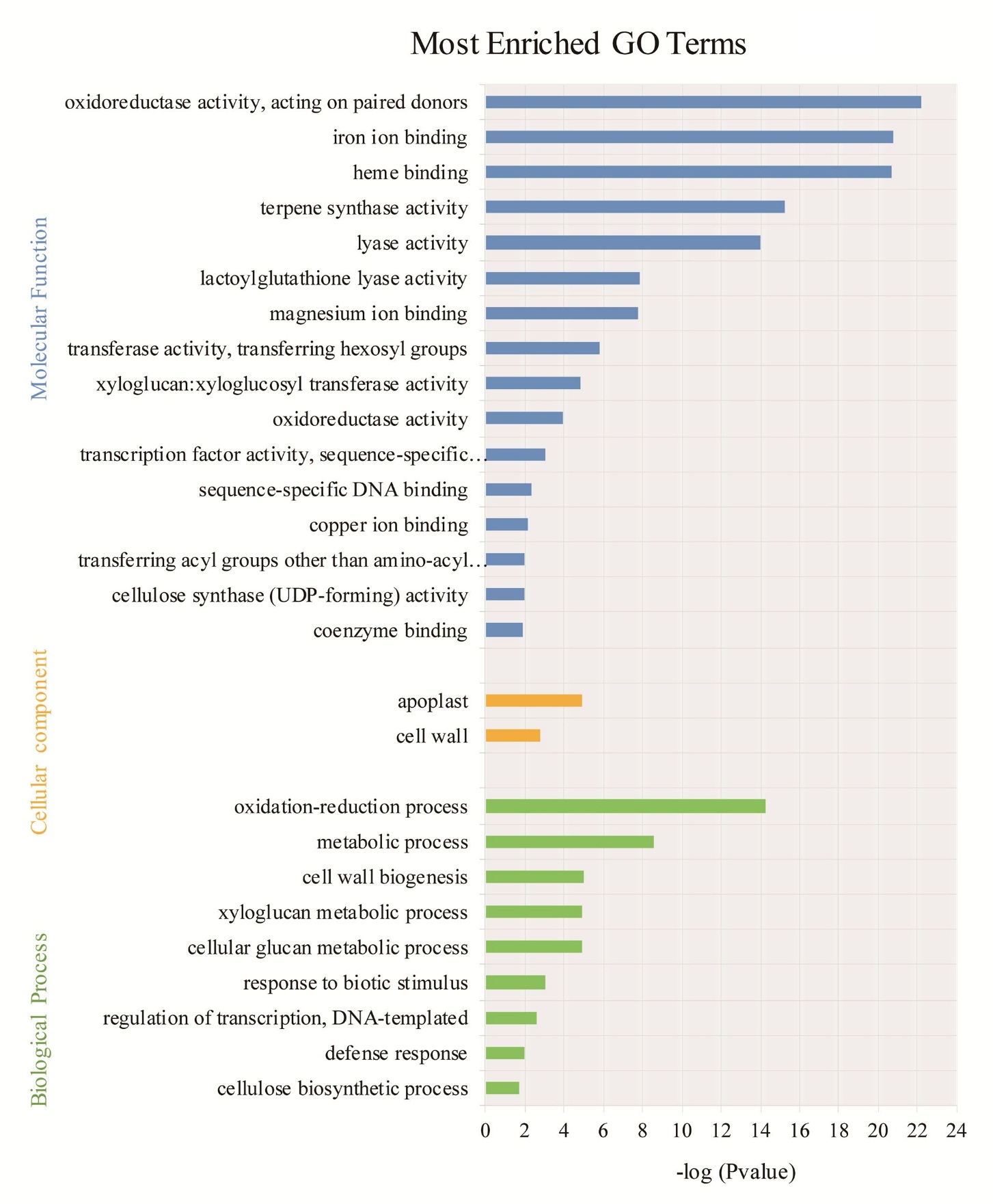


**Figure S2** GO analysis of DEGs in glandless cotton compared with glanded cotton.


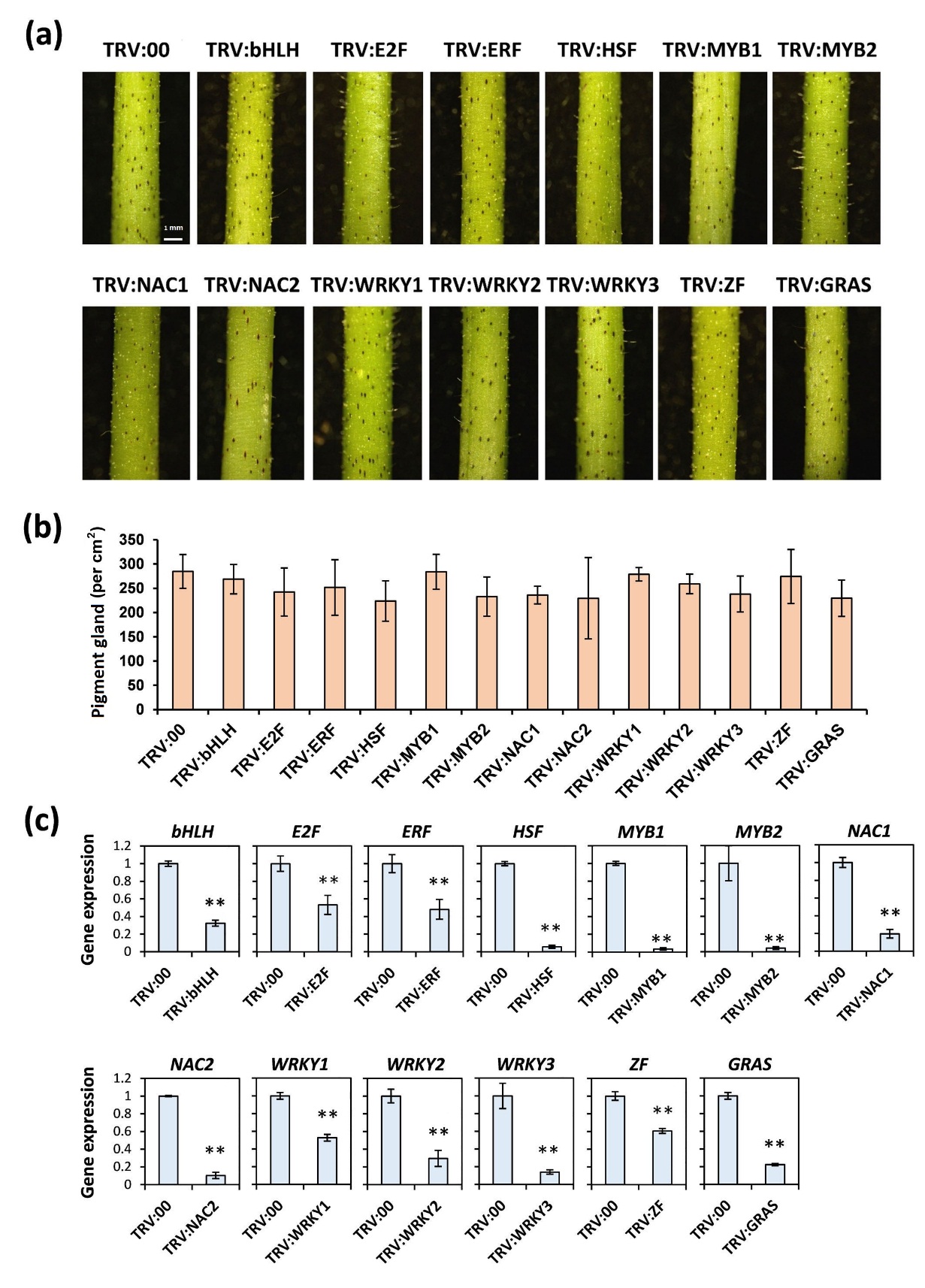


**Figure S3** Functional characterization of candidate TFs by VIGS in ‘TM-1’. (a) Phenotype of pigment glands on stems of control (TRV:00) and target gene-silenced plants (TRV:targets). (b) Pigment gland density in stems of control and target gene-silenced plants (n ≥ 15, ** *P* < 0.01, *t*-test). (c) Silencing efficiency of target *TF* genes were analyzed by qPCR (n ≥ 6, ** *P* < 0.01, *t*-test).


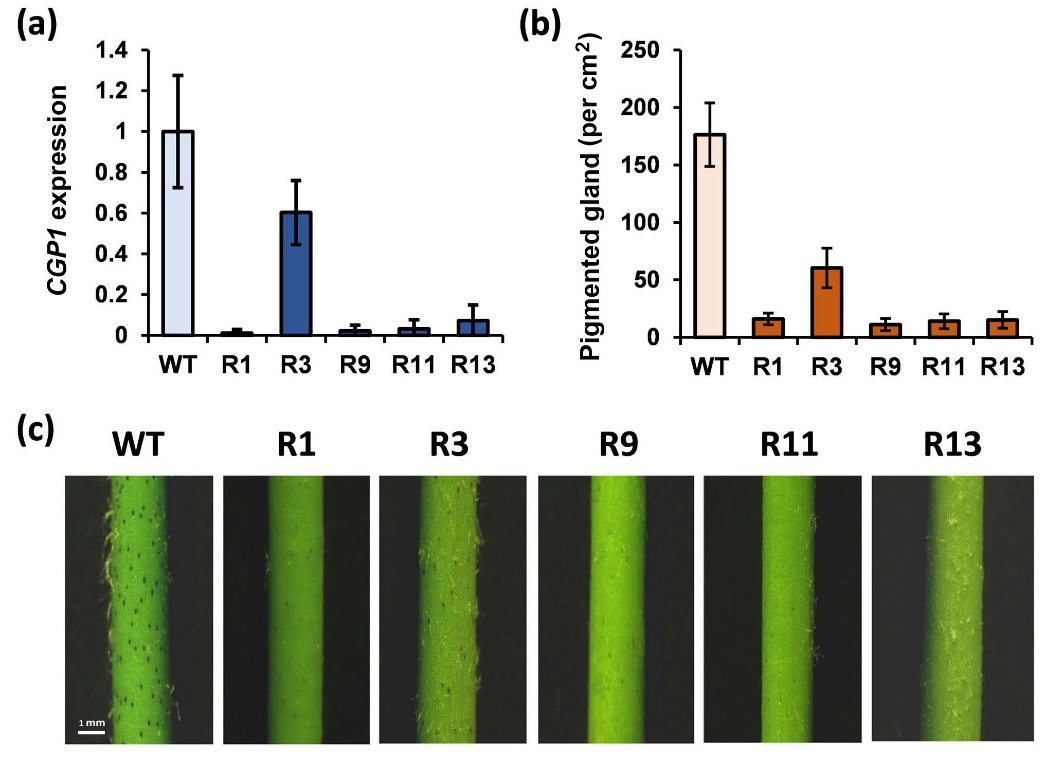


**Figure S4** RNAi-mediated silencing of *CGP1* in stably transformed cotton. (a) Silencing efficiency of *CGP1* in T_1_ transgenic plants (n ≥ 6, ** *P* < 0.01, *t*-test). (b) Pigmented gland density of WT and T_1_ transgenic plants (n ≥ 15, ** *P* < 0.01, *t*-test). (c) Phenotypes of WT and T_1_ transgenic plants.


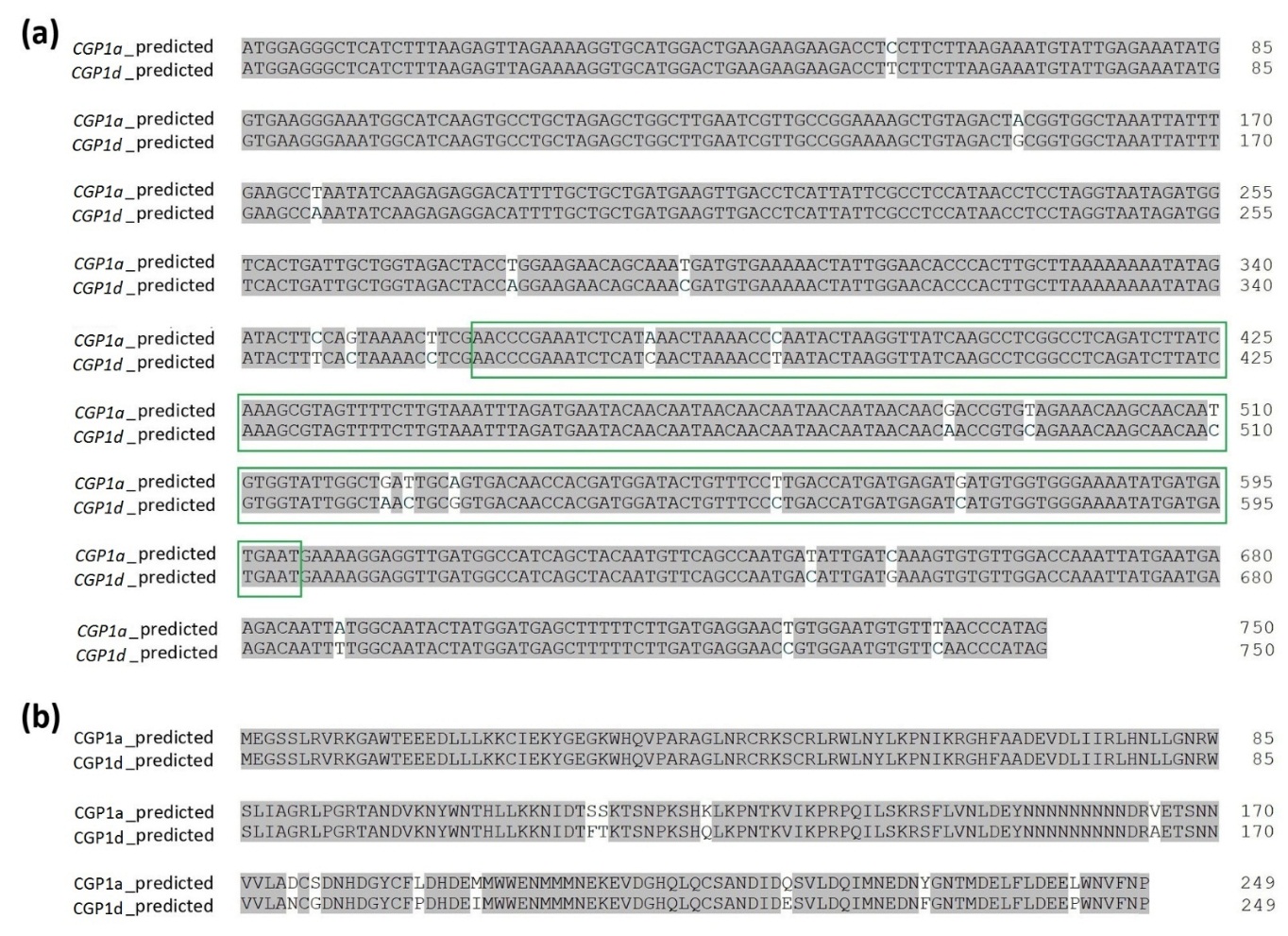


**Figure S5** Genome database sequences of CGP1a and CGP1d. (a) Nucleotide sequence alignment of *CGP1a* and *CGP1d*. The specific region of *CGP1* targeted by the RNA interference (RNAi) construct is boxed. (b) Amino acid sequence alignment of CGP1a and CGP1d. Conserved amino acids are indicated in grey.


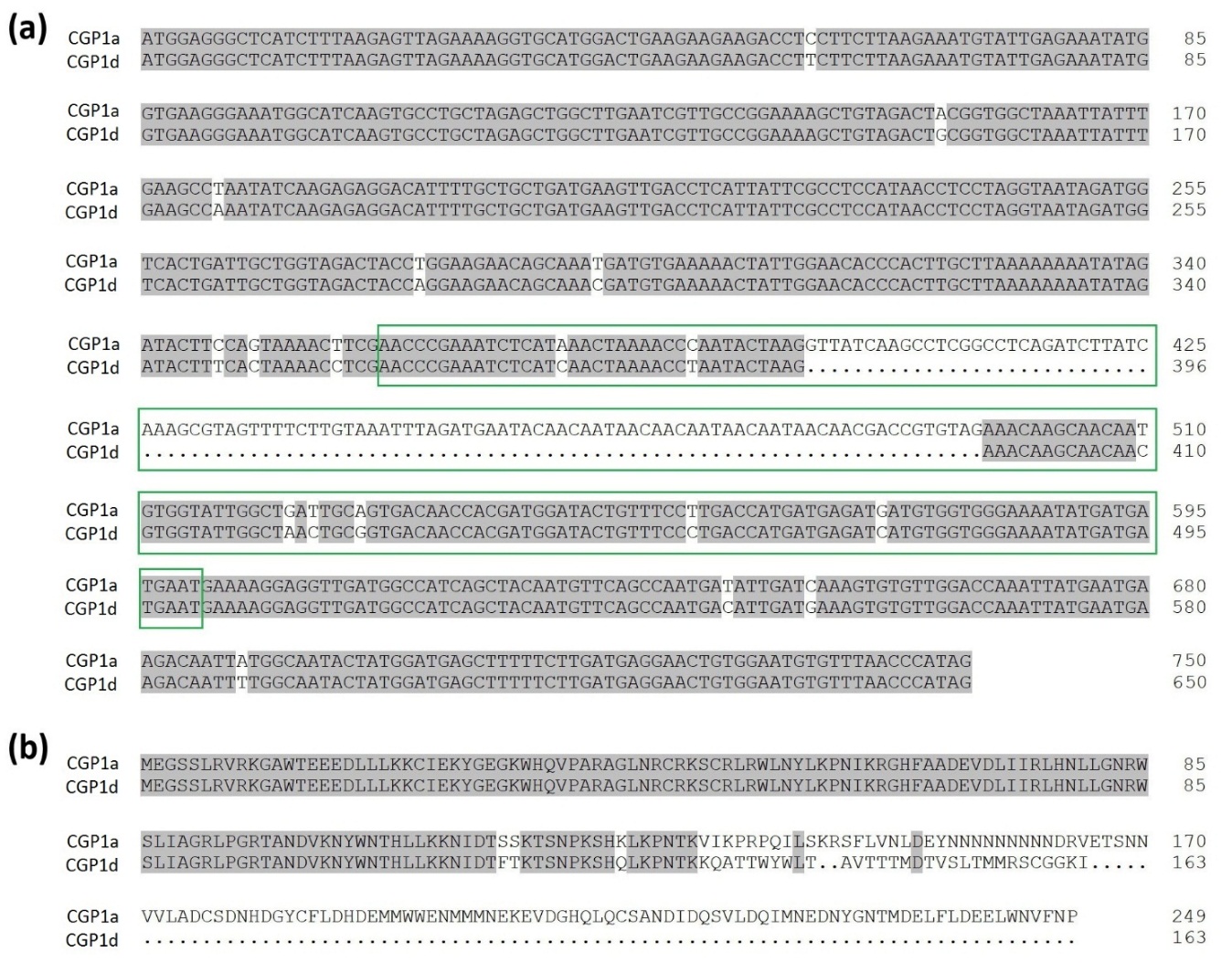


**Figure S6** Cloned CGP1a and CGP1d sequences. (a) Nucleotide sequence alignment *CGP1a* and *CGP1d*. The specific region of *CGP1* targeted by the RNA interference (RNAi) construct is boxed. (b) Amino acid sequence alignment of CGP1a and CGP1d. Conserved amino acids are indicated in grey.


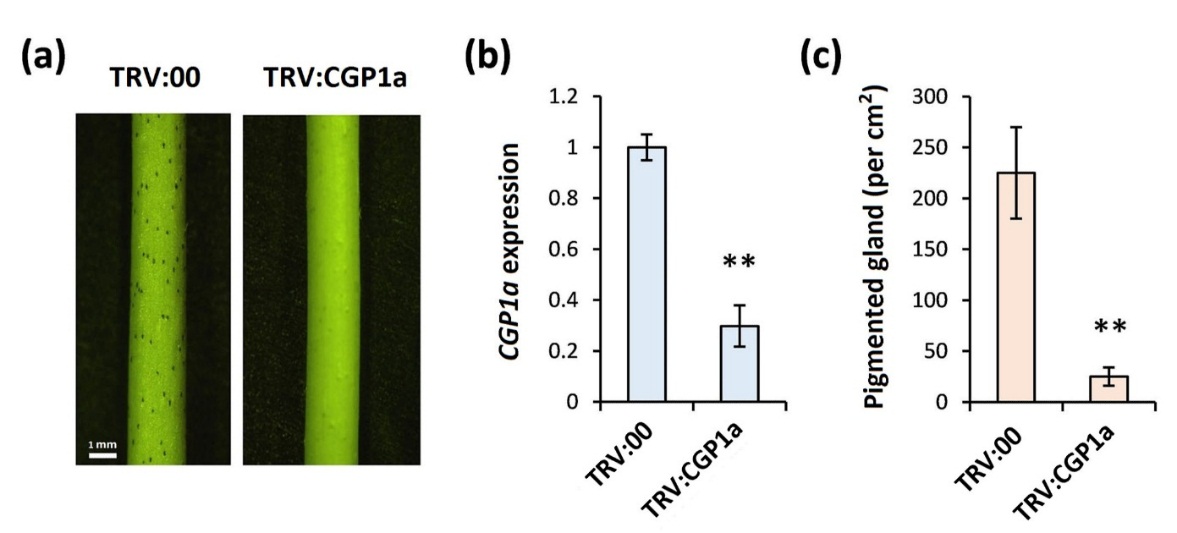


**Figure S7** Specific silencing of *CGP1a* in cotton. (a) Phenotypes of control (TRV:00) and *CGP1a*-silenced (TRV: CGP1a) cotton. (b) Silencing efficiency of *CGP1a* (n ≥ 6, ** *P* < 0.01, *t*-test). (c) Pigmented gland density of TRV:00 and TRV: CGP1a (n ≥ 15, ** *P* < 0.01, *t*-test).


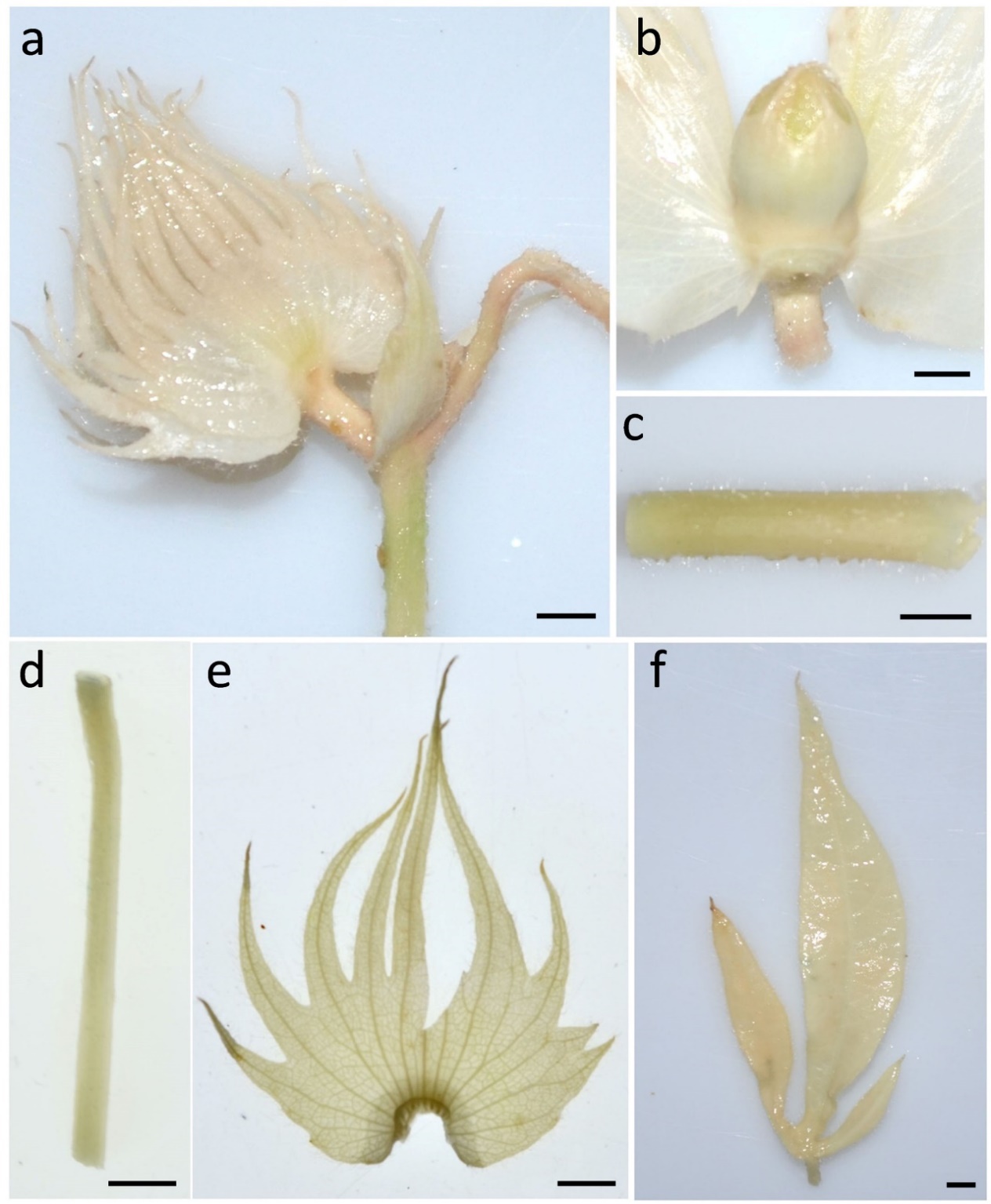


**Figure S8** GUS staining of the glandless cotton cultivar ‘YZ-1’ transformed with the ProCGP1-GUS construct (a, fruit-bearing branch; b, bud; c, stem; d, pedicel; e, bract; f, leaf), bar = 5 mm.


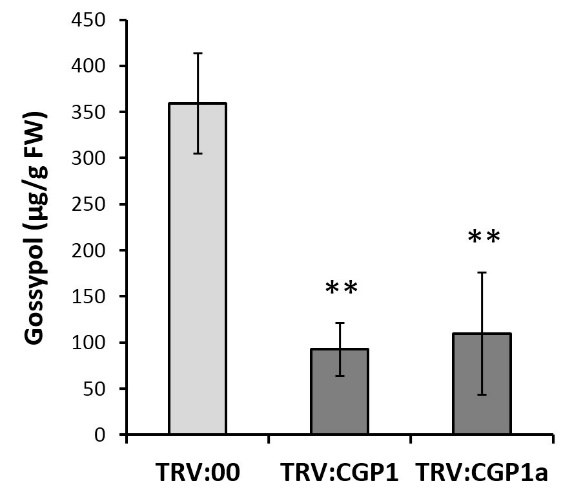


**Figure S9** Gossypol content in VIGS-silenced plants. Gossypol content in stems of control (TRV:00), CGP1-silenced (TRV: CGP1), and CGP1a-silenced plants (TRV: CGP1a) (n ≥ 10, ** *P* < 0.01, *t*-test).


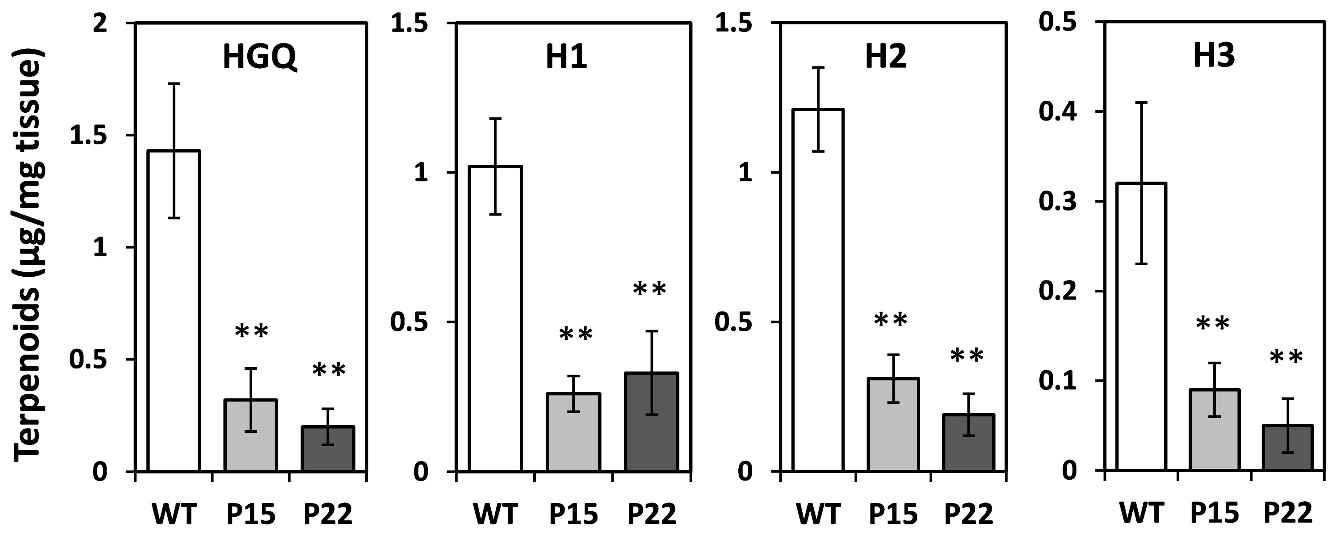


**Figure S10** Levels of gossypol-related terpenoids in WT and *cgp1* mutants. HGQ: hemigossypolon; H: heliocides. (n ≥ 6, ** *P* < 0.01, *t*-test).
